# Elicitor-Based Biostimulant PSP1 Protects Soybean Against Late Season Diseases in Field Trials

**DOI:** 10.3389/fpls.2018.00763

**Published:** 2018-06-12

**Authors:** Nadia R. Chalfoun, Sandra B. Durman, Jorge González-Montaner, Sebastián Reznikov, Vicente De Lisi, Victoria González, Enrique R. Moretti, Mario R. Devani, L. Daniel Ploper, Atilio P. Castagnaro, Björn Welin

**Affiliations:** ^1^Instituto de Tecnología Agroindustrial del Noroeste Argentino – Consejo Nacional de Investigaciones Científicas y Técnicas–Estación Experimental Agroindustrial Obispo Colombres, Las Talitas, Argentina; ^2^Bayer S.A., Argentina – Crop Science LATAM 2, Crop Science Research, Buenos Aires, Argentina; ^3^Asociación Argentina de Consorcios Regionales de Experimentación Agrícola, Buenos Aires, Argentina; ^4^Estación Experimental Agroindustrial Obispo Colombres, Las Talitas, Argentina; ^5^ANNUIT S.A., Buenos Aires, Argentina

**Keywords:** AsES, *Corynespora cassiicola*, *Septoria glycines*, *Cercospora kikuchii*, *Cercospora sojina*, crop protection

## Abstract

Currently, fungicide application in soybean production accounts for an important amount of global pesticide use, and it is therefore most desirable to find new healthier and more environmental friendly alternatives for the phytosanitary management in this crop. In this study, we present convincing evidence for effective induction of disease protection by the agricultural biostimulant PSP1, a formulation based on the plant-defense eliciting activity of the fungal protease AsES (*Acremonium strictum* elicitor subtilisin), in multiple field trials in Argentina.

PSP1 was shown to combine well with commercial spray adjuvants, an insecticide, a herbicide and fungicides used in Argentinian soybean production without losing any defense-inducing activity, indicating an easy and efficient adaptability to conventional soybean production and disease management in the region. Results from multiple soybean field trials conducted with different elite genotypes at several locations during two consecutive growing seasons, showed that PSP1 is able to induce an enhanced pathogen defense which effectively reduced late season disease (LSD) development in field-grown soybean. This defense response seems to be broad-range as disease development was clearly reduced for at least three different fungi causing LSDs in soybean (*Septoria glycines, Cercospora kikuchii* and *Cercospora sojina*). It was noteworthy that application of PSP1 in soybean alone gave a similar protection against fungal diseases as compared to the commercial fungicides included in the field trials and that PSP1 applied together with a fungicide at reproductive stages enhanced disease protection and significantly increased grain yields.

PSP1 is the first example of an elicitor-based strategy in order to efficiently control multiple fungal diseases under field conditions in the soybean crop. These results show the feasibility of using induced resistance products as complements or even full-good replacements to currently used chemical pesticides, fulfilling a role as important components of a more sustainable crop disease management system.

## Introduction

In the last two decades soybean [*Glycine max* (L.) Merrill] has become the most important crop in Argentina, both in terms of acreage and economic value. Currently, Argentina is the third largest soybean producer in the world with an annual production of around 60 million tons and the largest exporter of soybean derivative products, such as soybean oil and meal, responsible for the trading of almost half of these products in the world (Ploper, 2011). The expansion of soybean production areas in combination with no till management, to improve soil conservation, has impacted heavily on agro-ecosystems causing a substantial change in prevalence, frequency and intensity of soybean diseases in Argentina (Ploper et al., 2006). As a consequence, in the last two decades late season diseases (LSDs), caused predominantly by necrotrophic fungi, have become the most important pathologies affecting soybean production yields (Carmona et al., 2015). In the Pampas region, the most important soybean production area in Argentina, epiphytic LSDs include Septoria brown spot (SBS) (*Septoria glycines glycines* Hemmi), Cercospora leaf blight (LB) and purple seed stain [*Cercospora kikuchii* (Tak. Matsumoto and Tomoy) M. W. Gardner], and since 2009 frog-eye leaf spot (FLS) (*Cercospora sojina* Hara) (Carmona et al., 2015), while in the Northwest of Argentina soybean target spot (STS) caused by the fungus *Corynespora cassiicola* (Berk and M. A. Curtis) C. T. Wei., has become an increasingly important disease in later years (De Lisi et al., 2014). Currently, due to the lack of genetic resistance against most LSDs, the main control strategy for disease management consists in the application of foliar fungicides at reproductive stages (Ploper, 1999; Carmona, 2011).

Synthetic chemical pesticides have contributed significantly to the sizable increase in global agricultural production in recent decades (Hillocks, 2012), however, the overuse of these chemicals has primarily raised major concerns on the negative impact on human and animal health and the environment (Fantke et al., 2012) but has also dramatically increased crop production costs, especially in soybean (Lenssen, 2013). Consequently, there is a growing demand both from the society as well as producers for cheaper and more environmental-friendly alternatives to the chemical fungicides frequently used today for efficient disease control in crop production including soybean.

As a direct consequence of the important increase in pesticide use in the agriculture production, the European Union (EU) along with many other countries around the world has implemented more stringent regulations on registration of plant protection chemicals, and directed policies toward promoting non-hazardous biological alternatives (Hillocks, 2012; Skevas et al., 2013).

One promising strategy for crop disease management, which could supplement and/or replace chemical pesticides in the near future, is the application of biocontrol products that are capable of triggering innate plant defense responses. There are many examples in the literature of such elicitors or activators, involving compounds of both synthetic and natural origin, with capability to induce disease resistance in many plant species including soybean. The compound 1-aminocyclopropane-1-carboxylic acid (ACC), a precursor of ethylene biosynthesis; and 2,6-dichloroisonicotinic acid (INA), a molecule derived from salicylic acid (SA), efficiently induced resistance to the soybean fungal pathogens *Phytophthora sojae* and *Sclerotinia sclerotiorum*, respectively (Dann et al., 1998; Sugano et al., 2013). Benzothiadiazole (BTH) [benzo(1,2,3)thiadiazole-7-carbothioic acid *S*-methyl ester, also known as acibenzolar-*S*-methyl (ASM)], a structurally related functional analog of SA released commercially as Bion^®^ (Syngenta), has been shown to be effective against a wide range of soybean pathogens causing damaging soil-borne diseases such as brown stem rot, Fusarium wilt, Phytophthora root rot, Rhizoctonia root rot, and Sclerotinia stem rot (Dann et al., 1998; Faessel et al., 2008; Nafie and Mazen, 2008; Abdel-Monaim et al., 2012; Han et al., 2013; Sugano et al., 2013). Foliar application of BTH or saccharin (benzoic sulfimide), a metabolite of probenazole, have been shown to induce systemic resistance against soybean rust caused by *Phakopsora pachyrhizi* (Srivastava et al., 2011; Cruz et al., 2014). There is also evidence that the mineral nutrient silicon (Si) and fertilizers containing phosphite (phosphorous acid) (Thao and Yamakawa, 2009; Silva et al., 2011; Carmona et al., 2018) can enhance resistance against soybean diseases. Reductions in soybean rust were demonstrated following Si treatment in field and greenhouse studies (Lemes et al., 2011; Cruz et al., 2014) and hydroponic soybean plants supplied with Si exhibited strongly improved resistance to rust (Arsenault-Labrecque et al., 2012). Nolla et al. (2006) reported that soil Si applications reduce the incidence of FLS (*C. sojina*) and downy mildew [*Peronospora manshurica* (Naumov) Syd.]. In addition, components of fungal cell walls have been shown to induce defense responses in soybean such as various sized fragments of *N*-acetylchitooligosaccharides derived from fungal chitin (Day et al., 2001), β-1,6-1,3 heptaglucan from *Phytophthora megasperma* f. sp. *glycinea* (Sharp et al., 1984; Cheong et al., 1991) and an endopolygalacturonase from *S. sclerotiorum* (Zuppini et al., 2005), although only chitosan oligosaccharides effectively induces disease resistance in soybean plants (Prapagdee et al., 2007). An extract of giant knotweed (*Reynoutria sachalinensis*) formulated as the product Regalia^®^ (Marrone^®^ Bio Innovations, Inc., Davis, CA, United States) is claimed to control *C. kikuchii* on soybean (Su et al., 2011) and EplT4, a peptide from *Trichoderma asperellum* T4 induces a number of defense-related responses and protects plants against infection by *Cercosporidium sofinum* (Wang et al., 2013).

Although defense elicitors have been known for decades, relatively little information is available on more extended agronomic and disease protection studies for this kind of compounds in field conditions (Heil, 2014). In soybean for example, there are only three reports evaluating performance of elicitors in field conditions; INA and BTH (Dann et al., 1998), Si (Lemes et al., 2011) and BTH and humic acid (Abdel-Monaim et al., 2012), and only two of those assessed growth parameters and yield (Dann et al., 1998; Abdel-Monaim et al., 2012). Information from extensive field trials is vital if this type of bioproducts will eventually be considered as a viable alternative to chemical pesticides by farmers.

We have previously reported the characterization of the fungal extracellular protein AsES, a subtilisin-like protease secreted by the strawberry opportunistic pathogen *Acremonium strictum* strain SS71 (Castagnaro et al., 2012; Chalfoun et al., 2013). This fungal protein has the capability to induce an innate defense response and generate strong protection against anthracnose in strawberry (Chalfoun et al., 2013; Hael-Conrad et al., 2018) and gray mold in Arabidopsis (Hael-Conrad et al., 2015). In this work, we present evidence from multiple soybean field trials at several locations in the Pampas region in Argentina that a formulation containing AsES, PSP1, provides effective protection against several soybean LSDs when applied alone or in combination with commercial fungicides.

## Materials and Methods

### Production of PSP1 Product

*Acremonium strictum* W. Gams strain SS71 (DSMZ accession number DSM 24396) was cultured in soybean meal broth (SMB) containing 0.5% soybean meal, 0.05% KH_2_PO_4_, 0.05% K_2_HPO_4_, 0.02% MgSO_4_, 0.002% CaCl_2_, and 0.002% of a microelement solution (1.2% FeSO_4_.7H_2_O, 0.25% MnSO_4_.H_2_O, 0.025% CoCl_2_.6H_2_O, 0.25% ZnSO_4_.7H_2_O, 0.05% CuSO_4_.5H_2_O, 0.02% Na_2_MoO_4_.2H_2_O, 0.5% citric acid) supplemented with 1% (w/v) glucose and adjusted to pH 6.5. A 1.5 L-broth was autoclaved for 15 min in a 4 L-fermentation reactor and once the medium had cooled down to room temperature it was inoculated adding a conidia aqueous suspension of *A*. *strictum*, prepared from fungal colonies grown on Potato Dextrose Agar (PDA) plates, until reaching an initial concentration of 1 × 10^6^ conidia/ml of broth. Fungal fermentation was performed at 28°C with sterile air bubbling during 3 days. The culture broth was thereafter centrifuged at 9,000 × *g* for 15 min to separate fungal biomass from the medium, and the collected supernatant was adjusted to pH 5.5, followed by a membrane filtration using sterile 0.45 μm pore filters.

Proteolytic activity in the supernatant was measured by enzymatic hydrolysis of the chromogenic peptide *N*-Succinyl-Ala-Ala-Pro-Phe-*p*-nitroanilide (Suc-AAPF-*p*NA; Sigma) as described previously (Chalfoun et al., 2013). Briefly, 10 μl of the supernatant was diluted in 20 mM Tris-HCl (pH 7.5) to a final volume of 500 μl. After 2 min of preincubation of the reaction mixture without substrate at 37°C, 10 μl of 5 mM Suc-AAPF-*p*NA (final concentration of 0.1 mM) was added and the mixture incubated for 30 min under the same conditions. Reactions were stopped by addition of phenylmethylsulfonyl fluoride (PMSF) to a final concentration of 1 mM and the proteolytic activity was quantitatively assayed according to Moallaei et al. (2006). Total soluble protein (TSP) content in the supernatant was determined using the Bradford colorimetric assay with bovine serum albumin (BSA) as protein standard (Bradford, 1976). Production batches containing 25–30 U ml^-1^ of protease activity and 40–48 μg TSP ml^-1^ were chosen for lab assays and field trials. One unit of protease activity was defined as the release of 1 μmol of pNA per minute at 37°C and pH 7.5. This protocol corresponded to the optimized production method of the PSP1 product (unpublished results). All batch samples were stored at 4°C until further used.

### Plant Material and Growing Conditions

In general 10 soybean seeds from cv. A8000 RG (susceptible to STS disease) were sown into 4-L plastic pots with 3 kg of sterilized mix of washed sieved sand, commercial humus and soil (1:1:2). Pots were immediately watered with neutral oxyquinoline sulfate (0.5 g/L). Five days after seedling emergence, each pot was thinned to three seedlings at vegetative cotyledon stage (VC), corresponding to unifoliate leaves unrolled sufficiently so that the leaf edges are not touching (Fehr et al., 1971). Seedlings were regularly watered with deionized water and plants were grown in a greenhouse under natural light conditions with controlled air temperature (mean 20.2 ± 5.2°C ranging from 12.4°C at night to 33.0°C during the day) and a RH of 82 ± 14%. In spring and autumn, high pressure sodium lamps (400 W) adjusted to a 12-h photoperiod were used to supplement natural light, giving a light intensity of approximately 220 μmol photons m^-2^ s^-1^.

### Phytopathological Test of Soybean Target Spot (STS)

#### Growth Conditions of *C. cassiicola*

A pathogenic isolate of *C. cassiicola* (C4), obtained from symptomatic soybean leaves collected in the 2015 growing season at the locality of San Agustín (S 26° 49′30.43″; WO 64° 51′02.68″), Cruz Alta, Tucumán, Argentina, was used for all plant inoculations. Extensive morphological and molecular identification of *C. cassiicola* isolate C4 was performed prior to experimental use (data not shown). The C4 isolate was single-spore propagated to obtain pure cultures on PDA medium supplemented with 0.2% (v/v) lactic acid under continuous fluorescent light (165.3 μmol m^-2^ s^-1^) at 25 ± 2°C for 12 days. Pure colonies were preserved at 4°C using the Castellani’s method (Dhingra and Sinclair, 1995).

#### Inoculum Preparation of *C. cassiicola*

Disks of PDA-cultured isolate C4 of *C. cassiicola* were placed on Petri dishes and incubated in a growth chamber at 25 ± 2°C with an 18-h photoperiod of fluorescent light (52.7 μmol m^-2^ s^-1^) for 6 days. Fragments of fungal colonies were thereafter transferred to new Petri dishes and incubated in the same growth conditions for another 10 days before finally being placed under continuous white light (165.3 μmol m^-2^ s^-1^) for 2 days to stimulate conidia production.

Plate surface of 12-day-old fungal colonies, grown as described above, was carefully scraped with a sterile loop and suspended in distilled sterile water. The resulting fungal suspension was shaken vigorously for 15 min at 25°C and then filtered through sterile miracloth to remove mycelial debris. Conidia were counted using a cell-counting hemocytometer (Neubauer chamber) under an optical microscope and conidia concentration was adjusted to 5 × 10^4^ conidia/ml with sterile 0.02% Tween 20.

### Induced Resistance (IR) Bioassay Against STS

Bioassay of double treatment was performed on soybean plants at growth vegetative stage V3 corresponding to plants with three nodes on the main stem with fully developed leaves beginning with the unifoliate node (Fehr et al., 1971), following a procedure similar to that previously described (Salazar et al., 2007). First, aerial parts of plants were sprayed to run-off with each product to be evaluated as resistance-eliciting treatment (see details below), maintained under optimal soybean growth conditions, and after 3 days inoculated by foliar spraying to run-off with a conidial suspension of the virulent isolate C4 of *C. cassiicola* (5 × 10^4^ conidia/ml). A total of 5 ml of the conidial suspension prepared as described above was applied per plant as a fine mist using an atomizer on the adaxial surface of leaves. After inoculation, plants were maintained in an infection chamber at 28°C, 90% RH, and 12-h photoperiod under fluorescent light (700 μmol m^-2^ s^-1^). After 72 h in the infection chamber, plants were transferred to a plant growth cabinet for the duration of the experiment, where the natural photon flux density at the plant canopy height was approximately 700 μmol m^-2^ s^-1^, temperature held at 25 ± 2°C, and the RH was maintained at 80 ± 5% for the first 2 days using a misting system. Temperature and RH were monitored using a TH-508 thermohygrograph (Impac, Brazil).

#### Effect of Adjuvants on Defense Elicitor Effect of PSP1

Commercial adjuvants A1^1^, A2^2^, and A3^3^ were tested. A1 and A2 contain nonylphenol ethoxylates (NPEs) as active ingredient (a.i.) whereas A3 is mainly formulated with fatty acid methyl esters from vegetable oils (FAME). In addition, A1 contains chelating and acidifying agents whereas A2 is formulated with silicone and an anti-evaporation agent. Aqueous solutions of product PSP1 (adjusted to 0.5 U ml^-1^), combined with the surfactant agent 0.02% Tween 20 or the commercial adjuvants A1 (0.8 ml L^-1^), A2 (0.3 ml L^-1^) or A3 (3 ml L^-1^), were sprayed onto the canopy 3 days prior to inoculation with the pathogenic strain C4 of *C. cassiicola*.

#### Compatibility Assays of PSP1 With Commercial Agrochemicals

To analyze the possible co-application of PSP1 with registered agrochemicals for soybean production, plants were treated with PSP1 (adjusted to 0.5 U ml^-1^) in combination with the following commercial products plus the adjuvant recommended by the manufacturer: insecticide (I) at the final concentration 0.75 ml/L (dilution 1:10 of field recommended rate (a.i.: 10 g/L imidacloprid and 1.25 g/L lambda-cihalotrin) plus adjuvant A2 (0.3 ml L^-1^); herbicide (H) diluted to 1.5 ml/L (dilution 1:10 of field recommended rate) (a.i.: 662 g/L glyphosate) plus adjuvant A2 (0.3 ml L^-1^) and finally fungicide F1 (a.i. 375 g/L trifloxystrobin and 160 g/L cyproconazole) diluted to 0.015 ml/L (dilution 1:100 of field recommended rate) combined with the adjuvants A2 or A3 at the concentrations mentioned above. These mixtures were applied on foliage of soybean plants by spraying to run-off 3 days prior to inoculation with the virulent isolate C4. Tested concentrations showed no phytotoxic effect on soybean plants grown in controlled conditions, nor did they exhibit *in vitro* inhibitory activity against the isolate *C. cassiicola* C4 (data not shown).

#### Experimental Design in Soybean IR Assays and STS Assessment

Normal disease development was monitored in pathogen control plants, firstly sprayed with water (mock-treated) and 3 days later inoculated with the pathogenic strain C4 of *C*. *cassiicola* (P). A complete randomized experimental design was performed with nine plants (biological replicates) for each treatment and each experiment was carried out twice. STS disease severity was evaluated on the third and fourth trifoliate leaves (soybean growth stages V3 and V4) 4, 7, and 10 days post-inoculation (dpi) using a standard area diagram set (Soares et al., 2009). Covered lesion areas (%) were adjudicated to the following disease severity classes: 1 (0–1%), 2 (2–10%), 3 (11–20%), 4 (21–50%), and 5 (≥50%).

STS disease severity index (DSI) was calculated for each plant from the scores of the six leaflets of each plant and for each IR-treatment, and the value was expressed as a percentage using the formula below.

$$\text{STS DSI}(\%)=\Sigma \left(\frac{Ax0+Bx2+Cx11+Dx21+Ex50}{Tx50}\right)\times 100;$$

where, A, B, C, D, and E are the number of leaflets corresponding to the numerical grades 1, 2, 3, 4, and 5, respectively, and T is the total number of leaflets multiplied by the maximum severity grade 5, where T = A+B+C+D+E. A STS DSI of 0 was given to plants where no disease was present, and 100 to plants where all leaflets were assigned a score of 5.

Data from the STS DSI evaluation at 4, 7, and 10 dpi were used to calculate the area under disease progress curve (AUDPC) (Madden et al., 2007) for each plant and each IR-treatment according to the method of Shaner and Finney (1977).

#### Data Statistical Analysis

Statistical analyses were performed using the software Minitab V17. Treatment effects were assessed by Analysis of Variance (ANOVA). Factorial ANOVA test was done with data of two assays, considering experiment, treatment and their interaction as main effects. Means of STS DSI at each time and AUDPC were compared among treatments by Tukey’s HSD test at the 0.05 significance level. Dunnett’s test (*P* < 0.05) was used to compare each treatment to control treatment.

### Soybean Field Trials

(i) Tandil field trial: A single soybean field trial was conducted in the 2013/14 growing season in the town of Tandil located in the South of the Province of Buenos Aires, Argentina (Supplementary Figure 1). Certified seeds of the soybean variety LDC 3.7 were used. The trial was a randomized block design with an individual plot size of 8 m × 3 m, using three plots per treatment. Plots received standard soybean crop management without foliar fungicide application. The only fungicides applied in the trial were those included in the experimental design as detailed below. All experimental treatments were performed when plants reached the reproductive phenological stage R3 corresponding to beginning of pod as described by Fehr et al. (1971), (pod 3/16 inch long at one of the four uppermost nodes on the main stem with a fully developed leaf). Treatments were untreated control, PSP1, fungicide F1 (see above), fungicide F2 (a.i. 150 g/L trifloxystrobin and 175 g/L prothioconazole) or a mixture of PSP1 and fungicides. PSP1 was applied at 2,000 ml/ha, F1 at 150 ml/ha and F2 at 400 ml/ha and treatment of PSP1 combined with a fungicide was performed spraying soybean crop with a single application broth containing both products. Application broths were prepared with the adjuvant A3 (300 ml/100 L) and applied by spraying with a backpack sprayer at a rate of 100 L/ha.

Disease symptoms (incidence and severity) of SBS and frogeye leaf spot (FLS), caused by the two necrotrophic fungi *S. glycines* and *C. sojina*, respectively, were assessed when field-grown plants reached reproductive phenological stages R3 (beginning pod), R5 (beginning seed), and R6 (full seed). Plants in stage R5 contain seed 1/8 inch long in the pod at one of the four uppermost nodes on the main stem with a fully developed leaf, whereas those in R6 contain stage pod containing green seeds that fills the pod cavity at one of the four uppermost nodes on the main stem with a fully developed leaf (Fehr et al., 1971). All plots were harvested at the end of the trial and grain yield (expressed as kg/ha at 87% dry matter content) and 1,000-seed weight (TSW) were determined.

(ii) Pampas region field trials: Multiple soybean field trials were conducted in the 2014/15 growing season at 14 locations distributed in the Provinces of Córdoba (CB; three trials), Santa Fe (SF; five trials), Buenos Aires (BA; five trials) and Entre Ríos (ER; one trial), all belonging to the most important soybean production area of Argentina. Supplementary Figure 1 shows the geographic localization of all 14 trial sites. All trials were conducted with a randomized block design with an individual plot size of 3 m × 10 m, using five plots per treatment. Plots received standard soybean crop management without foliar fungicide application. The only fungicides applied in the trials were those included in the experimental design as detailed below. Trial treatments were sprayed when plants had reached phenological stages V6 (plants with six nodes on the main steam with fully developed leaves beginning with the unifoliate node) and R3 (Fehr et al., 1971). Treatments included in the trials were control with water at V6 and R3 (Control), PSP1 at V6, fungicide F1 at R3, PSP1 at V6 and F1 at R3 (PSP1-F1), and PSP1 plus F1 at R3 (PSP1+F1). Each foliar application was performed at a dose of 2,000 ml/ha for PSP1 and 150 ml/ha for F1. Application broth for all treatments was prepared with the adjuvant A3 (300 ml/100 L) and applied with a backpack sprayer calibrated to deliver 150 L/ha. Disease severity of LB (*C. kikuchii*) and SBS (*S. glycines*) were assessed 20 and 40 days after R3 applications. Plots were harvested at the end of the trial and yields expressed as kg/ha at 87% dry matter content.

Trials at different locations were divided into three groups according to the yield obtained for control plots at each site. High (H) yield trials were those having control treatments with yields greater than 4,500 kg/ha (three trials), medium (M) yield trials were those in which control treatments reached between 3,000 and 4,500 kg/ha (seven trials) and low (L) yield trials, which had control treatments with yields lower than 3,000 kg/ha (four trials). After classification into three groups according to yield, severity of SBS (*S. glycines*) and LB (*C. kikuchii*) were analyzed for each yield group. **Table 1** indicates the province, location and soybean variety of all the trials.

**Table 1:** Characteristics of soybean trials categorized in low, medium, and high yield.

Low yield (L)	Trial	E14	E15	E30	E32			
	Variety	DM3700	DM4670	DM4990	DM3810			
	Province	BA	BA	SF	SF			
	Location	Villegas	Necochea	Santa Isabel	San M. de las Escobas			

Medium yield (M)	Trial	E1	E3	E17	E19	E26	E34	E35
	Variety	DM4612	DM3810	DM4990	NA6448	NA5009	LDC5.9	NA5909
	Province	BA	BA	BA	CB	ER	SF	SF
	Location	Fontezuela	Pehuajó	La Dulce	Colazo	Gualeguaychu	Rafaela	San Jerónimo Norte

High yield (Y)	Trial	E20	E22	E36				
	Variety	DM4210	DM4990	NA5909				
	Province	CB	CB	SF				
	Location	Córdoba	Cañada de Luque	Fuentes				


#### Data Analysis of Soybean Field Trials

Statistical analyses were performed using the software Minitab V17. Treatment effects were assessed by Analysis of Variance or by Welch ANOVA (when homoscedasticity was not supported). Means among treatments were compared correspondingly by Tukey’s HSD test or Paired Games-Howell test at the 0.05 significance level. Dunnett’s test was used to compare each treatment to control treatment. Data of SBS and FLS severity and incidence collected from the field trial in Tandil during the 2013/14 growing season were not sufficient to perform compelling statistical analysis.

## Results

### Effect of Surfactant Adjuvants on PSP1 Activity

One of the most critical parameters determining the efficiency of a biocontrol product is the application method as it needs to be easy to handle and efficiently adhere to the plant tissue for a sufficient period of time to successfully carry out its activity. As part of the technological development of PSP1, the evaluation of different commercial spray adjuvants commonly used in soybean foliar applications containing different surfactants (surface active agents) was undertaken and compared to the ionic surfactant Tween 20.

**FIGURE 1:**
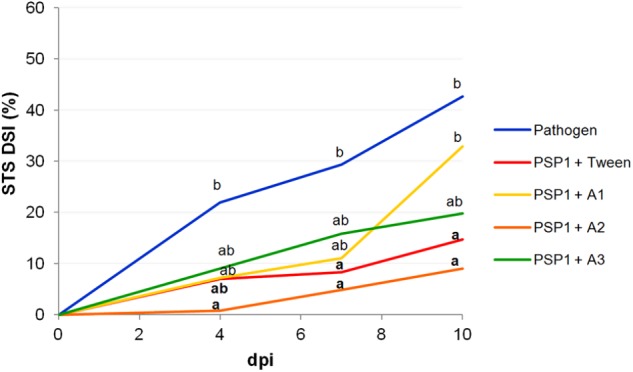
Effect of surfactant adjuvants on the defense-eliciting activity of PSP1 against *C. cassiicola* in soybean. Aqueous solutions of PSP1 (0.5 U ml^-1^), combined with Tween 20, A1 [nonylphenol ethoxylates (NPEs) + chelating and acidifying agents], A2 (NPEs + silicone and anti-evaporation agent) and A3 [fatty acid methyl esters (FAME)], were sprayed on soybean plants grown under controlled conditions 3 days prior to inoculation with the virulent strain C4 of *C. cassiicola* (5 × 10^4^ conidia/ml) and induced resistance against soybean target spot (STS) was determined. Nine biological replicates (potted plants) were assessed for each treatment and the experiment was carried out twice. Factorial ANOVA test indicated no significant differences between the two experimental repetitions (*P* > 0.05) and results from one representative experiment are shown. STS severity was evaluated on V3 and V4 trifoliate leaves of soybean plants treated with a product or mock (pathogen control) as the percentage of leaf area covered with disease symptoms and calculated as disease severity index (DSI) at 4, 7, and 10 days post-inoculation (dpi). Mean values of DSI at different time points are reported for each treatment calculated from one representative experiment with nine biological replicates. Values followed by different letters are significantly different according to Tukey’s HSD test (*P* < 0.05). Bold letters indicate statistically significant differences in STS protection of PSP1-treated plants as compared to mock-treated soybean plants, both infected with pathogenic strain C4 (Dunnett’s test; *P* < 0.05).

Protection against STS disease in plants treated with PSP1 combined with different adjuvants in a controlled growing environment is shown in **Figure 1** and Supplementary Table 1. Results indicate that the defense-eliciting activity of PSP1 slightly varied depending on the adjuvant added, although all three commercial adjuvants tested were compatible with PSP1. Adjuvants A2, A3 and Tween 20 were able to potentiate/maintain the eliciting activity of PSP1, whereas the disease protecting effect of PSP1 was slightly reduced when combined with A1. As a result of this study spray adjuvants A2 and A3 were chosen for further greenhouse studies and field trials.

### PSP1 Compatibility With Commercial Agrochemicals

**FIGURE 2:**
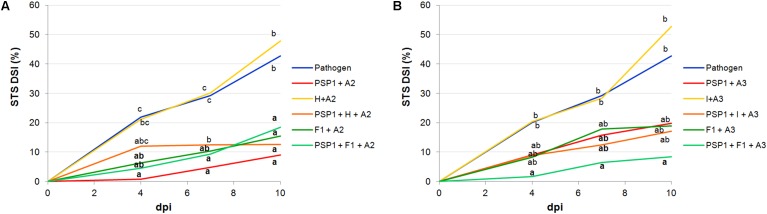
PSP1 compatibility with commercial agrochemical products: insecticide (I), herbicide (H) and fungicide (F1) combined with different adjuvants. The tested mixtures with adjuvant A2 are shown in **(A)**: PSP1+A2, H+A2, PSP1+H+A2, F1+A2 and PSP1+F1+A2; and those with adjuvant A3 are shown in **(B)**: PSP1+A3, I+A3, PSP1+I+A3, F1+A3 and PSP1+F1+A3. These mixtures were applied in soybean plants grown under controlled conditions 3 days prior to inoculation with the virulent strain C4 of *C. cassiicola* (5 × 10^4^ conidia/ml) and induced resistance against sobean target spot (STS) was determined. Nine biological replicates (potted plants) were assessed for each treatment and the experiment was carried out twice. Factorial ANOVA test indicated no significant differences between the two experimental repetitions (*P* > 0.05) and results from one representative experiment are shown. STS severity was evaluated on V3 and V4 trifoliate leaves of soybean plants treated with a product or mock (pathogen control) as the percentage of leaf area covered with disease symptoms and calculated as disease severity index (DSI) at 4, 7, and 10 days post-inoculation (dpi). Mean values of DSI at different time points are reported for each treatment calculated from one representative experiment with nine biological replicates. Values followed by different letters are significantly different according to Tukey’s HSD test (*P* < 0.05). Bold letters indicate statistically significant differences in STS protection of PSP1-treated plants as compared to mock-treated soybean plants, both infected with pathogenic strain C4 (Dunnett’s test; *P* < 0.05).

To study the compatibility of PSP1 with commercial agrochemical products routinely applied in soybean production, IR assays were carried out in soybean plants treated with an herbicide (H) (commercial formulation of glyphosate) and an insecticide (I) each combining with the adjuvants recommended by the manufacturer (A2 for H and A3 for I). For both formulations, concentrations were reduced to ensure that they did not exhibit any antifungal activity *per se in vitro* or phytotoxicity under controlled growing conditions (data not shown). Results showed that neither product had a negative effect on the defense-eliciting activity of PSP1 (**Figure 2** and Supplementary Table 2) and that a slight increase in protection was observed when the insecticide (I) was applied together with PSP1 and A3.

In addition, the disease protective effect of PSP1 combined with the commercial fungicide F1 supplemented with adjuvants A2 (**Figure 2A**) or A3 (**Figure 2B**) was studied (AUDPC values are shown in Supplementary Table 2). The F1 concentration used in the experiment was slightly lower than the minimal *in vitro* inhibitory concentration (MIC) against *C. cassiicola* C4 (data not shown). Results obtained for both adjuvants (A2 and A3) combined with F1 indicated that a higher residual fungicide activity was obtained with A2 (**Figure 2A**), in concordance with results obtained when adjuvants A2 and A3 were mixed with PSP1 alone, even though A3 is the adjuvant recommended by the F1 manufacturer. However, when F1 was added to the mixture of PSP1 plus an adjuvant the best protection was seen for F1, PSP1 and A3 (**Figure 2B**), indicating that adjuvant A3 could enhance the PSP1 elicitor activity as compared to A2 in the mixture of three components.

### PSP1 Efficiency on LSDs Management in Soybean

To analyze the effect of PSP1 application on LSDs development under standard soybean production practices, field trials at different locations in Argentina were performed during two growing seasons (2013-2015). During 2013/14 one exploratory trial at one geographical location was conducted to obtain preliminary information on PSP1 performance in the field and its competitiveness with leading market commercial fungicides, whereas in 2014/15, a total of 14 independent trials at different locations and agro-ecological conditions were aimed at showing the applicability of the product in commercial soybean production.

#### Field Trial in Tandil During Growing Season 2013/14

**FIGURE 3:**
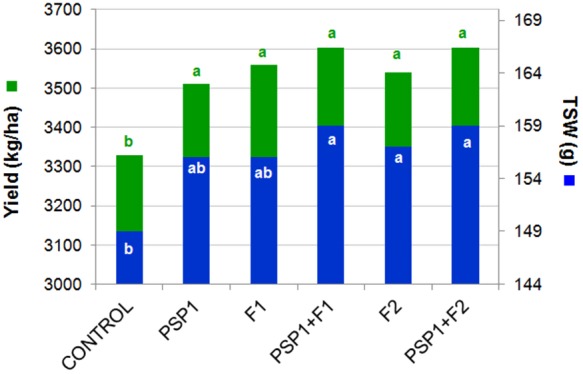
Soybean field trial in Tandil during growing season 2013/14. Yield (kg/ha) and TSW (g) were determined. All treatments were performed when soybean crop had reached the reproductive phenological stage R3. Control plants received standard soybean crop management without foliar fungicide application. F1 dosage = 150 ml/ha, F2 dosage = 400 ml/ha and PSP1 dosage = 2,000 ml/ha. Different letters in each column indicate statistically significant differences (according to Tukey’s HSD test; *p* < 0.05).

The first soybean field trial conducted in Tandil during the 2013/14 growing season compared the performance of PSP1 alone and in combination with commercial fungicides. As can be seen in **Figure 3** all treatments with PSP1, fungicides and combinations of the two products significantly improved yield and seed size (TSW) in a similar proportion when compared to non-treated control plants. In addition another growth parameter, plant height at harvest, was notoriously increased in all treatments as compared to control plants (data not shown).

**FIGURE 4:**
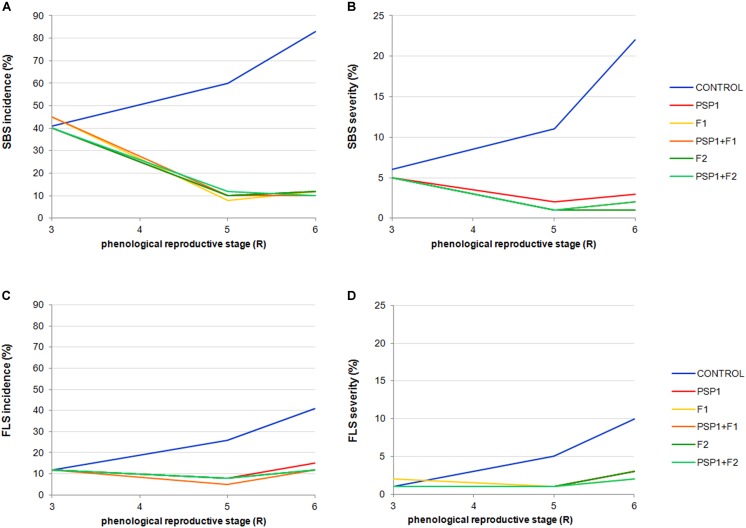
Soybean field trial conducted in Tandil during growing season 2013/14. Incidence and severity of SBS evaluated at phenological stages R3, R5, and R6 are shown in **(A,B)**, respectively. Incidence and severity of FPS evaluated at phenological stages R3, R5, and R6 are shown in **(C,D)**, respectively. Treatments were control (received standard soybean crop management without foliar fungicide or biocontrol treatment), PSP1, fungicide F1, fungicide F2, PSP1 plus fungicide F1 and PSP1 plus fungicide F2. The collected data, although highly informative, was not sufficient to perform a thorough statistical analysis.

Furthermore, although data collected was not sufficient for executing compelling statistical analysis, **Figure 4** show that application of PSP1, either of the two fungicides or a mixture of PSP1 and a fungicide, seemed to be equally effective in reducing SBS and FLS severity and incidence. The high similarity in disease protection among treatments strongly supports the yield data obtained for the field trial.

#### Field Trials at 14 Different Locations in the Pampas Region of Argentina During Growing Season 2014-15

**FIGURE 5:**
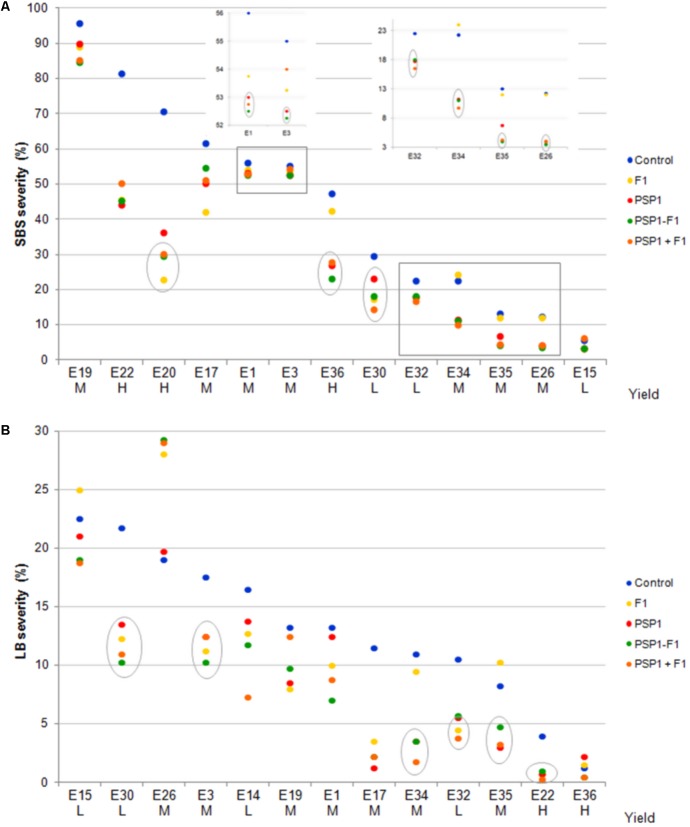
Soybean field trials at 14 different locations in the central region of Argentina during growing season 2014-15. Severities (%) of SBS (*S. glycines*) and LB (*C. kikuchii*) assessed 40 days after R3 application are shown in **(A,B)**, respectively. Trials (named E) are ranked according to decreasing severity values in the control treatment for each disease and classified according grain yield in low (L), medium M and high yield (H). Treatments were control (standard soybean crop management without foliar fungicide or biocontrol treatment), F1 (fungicide F1 alone at R3), PSP1 (PSP1 alone at V6), PSP1-F1 (PSP1 at V6 plus fungicide F1 at R3 one at the time) and PSP1+F1 (PSP1 plus fungicide F1 at R3 at the same time). Symbols in gray circles indicate treatments significant different to the correspondent control treatment (according to Dunnet test; *p* < 0.05) and gray boxes are enlarged on the inserted graphics for better view. SBS or LB severity were not evaluated for E14 and E20, respectively. L, low yield; M, medium yield; H, high yield.

The encouraging results from the field trial performed during the 2013-14 growing season prompted us to conduct a more extended study with an increased number of trials at different locations in order to obtain sufficient data for statistical analyses on LSDs disease protection and yield. We also wanted to test which application combination of PSP1 and fungicide that gave the best disease protection, applied separately in different phenological stages or combined in the same stage. Two important LSDs in this region, SBS and LB, were selected and disease severities were assessed 20 and 40 days after treatments in all trials. In **Figure 5** and Supplementary Table 3, trials (named E) are ranked according to decreasing severity values in the control treatment (standard soybean crop management without foliar fungicide or biocontrol treatment) for each disease and classified according to grain yield in low (L), medium (M) and high yield (H).

In all trials except one (E26 for LB), disease severity was reduced by at least one of the treatments and in 11/13 (for SBS) and 9/13 (for LB) of trials, all treatments showed a diminished disease severity. The most successful treatments were the applications of PSP1 alone or applied with F1 at different growth stages (PSP1-F1) or at the same time (PSP1+F1) where disease severity reduction was seen in 25 of the 26 studies, whereas F1 alone caused such effect in 21 out of the 26 trials. Among these trials, treatment with F1 alone, PSP1 alone or mixtures of both products (PSP-F1 or PSP+F1) significantly decreased disease severity as compared to control plants in 7, 13 and 14 cases of trials, respectively according to Dunnet test (highlighted by gray circles in **Figure 5** and bold letters in Supplementary Table 3). Statistical analysis is shown in Supplementary Table 3.

More importantly, the positive effect on disease protection was accompanied by an increase in yield for all treatments studied (Supplementary Table 4). Both the double treatment of PSP1 and F1 (PSP1-F1) as well as the treatment with PSP1 at V6 and fungicide at R3 increased the yield in all 14 trials, whereas F1 and PSP1 alone increased yield in 12/14 (86%) and 10/14 (71%) of trials, respectively. When analyzing the overall gain in yield we found that the highest increase was found for the two double treatments where 13% increments were observed, followed by fungicide application of 10% and PSP1 of 4%, which correlate well with the number of trials that generated increased yield for the different treatments.

**FIGURE 6:**
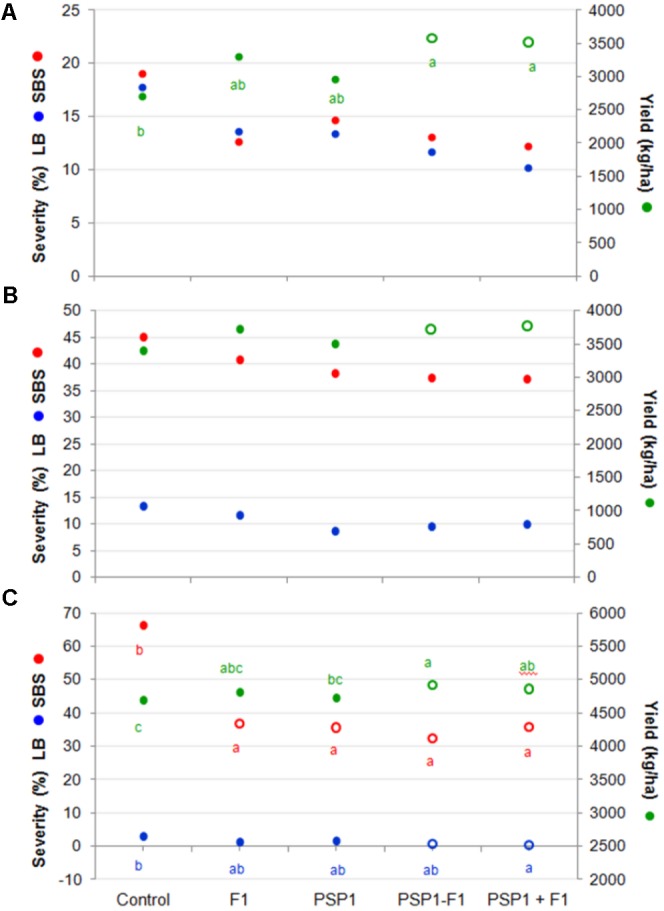
Soybean field trials at 14 different locations in the central region of Argentina during the 2014-15 growing season. Trials were divided in **(A)** low yield (L; < 3,000 kg/ha), **(B)** medium yield (MY; >3,000 and <4,500 kg/ha), and **(C)** high yield (H; >4,500 kg/ha). SBS severity (%), LB severity (%) and yield (kg/ha) in each subset of trials were evaluated. Treatments were control (received standard soybean crop management without foliar fungicide or biocontrol treatment), F1 (fungicide F1 at R3), PSP1 (PSP1 alone at V6), PSP1-F1 (PSP1 at V6 plus fungicide F1 at R3) and PSP1+F1 (combined application of PSP1 plus fungicide F1 at R3). Letters indicate significant differences among treatments (according to Tukey’s HSD test; *p* < 0.05). Hollow symbols indicate treatments demonstrating significant statistical difference compared to the control treatment (according to Dunnet test; *p* < 0.05).

Field trials were classified, as mentioned earlier, into three categories namely, high (H; > 4,500 kg/ha), medium (M; 3,000–4,500 kg/ha) and low yield (L; < 3,000 kg/ha) in order to evaluate if there were any noticeable differences among treatments in a high yield trial as compared to ones with lower yields. **Figure 6** shows that grain yield increased for all treatments and in all categories (H, M, and L yield trials) but the only treatment showing a statistically significant difference for all three categories was the mixture PSP1 and fungicide F1 applied simultaneously (PSP1+F1) at R3.

In accordance with results from the 2013-14 growing season, there is an inverse correlation between disease development and yield increase. Interestingly, SBS was found to be much more important in H yield trials (65%) with a very pronounced reducing effect (∼50%) when plants were treated with PSP1, fungicide or a mixture of both. In contrast, in the M and L yield trials with an intermediate and low SBS severity of 45% and 20%, respectively, these treatments were much less successful in reducing the disease severity (∼10%) as compared to H yield trials.

Another interesting observation was the reversed severity of LB (*C. kikuchii*), a close relative of the FLS pathogen *C. sojina*, where a very low disease frequency was detected in trials with H yield (3%), an intermediate in those with M yield (14%), and the highest in L yield trials (18–19%). Again there was a correlation of LB severity and yield as both treatments with PSP1 plus fungicide were the most effective in reducing LB development as well as producing the highest yields (**Figure 6**).

## Discussion

In this work, we have presented results showing the usefulness of the novel biostimulant, PSP1, based on the protease elicitor AsES, to control soybean LSDs both under greenhouse and field conditions. There are only a short number of reports demonstrating effective control of soybean diseases by foliar application of elicitor-based products (Dann et al., 1998; Prapagdee et al., 2007; Nafie and Mazen, 2008; Lemes et al., 2011; Han et al., 2013; Cruz et al., 2014) and there are only two cases, where elicitor-treatments has been described to be effective against necrotrophic pathogens causing soybean LSDs; i.e., soil Si applications reducing the incidence of FLS and downy mildew diseases (Nolla et al., 2006) and Regalia^®^-treatment controlling LB caused by *C. kikuchii* (Su et al., 2011).

One very important aspect to consider when developing a novel biocontrol product is the importance of its compatibility with commonly used agrochemicals in conventional crop production. The reason behind this is to minimize the amount of field applications to lower costs and environmental impact when applying the product, but also to facilitate acceptance of the new product by the farmer as its use will not significantly change crop management routines. Therefore, to preliminary assess the applicability and compatibility of PSP1 with agrochemicals commonly used in soybean production in Argentina we first conducted IR studies of target spot disease with different commercial spray adjuvants, an insecticide and the herbicide glyphosate under controlled greenhouse conditions. Interestingly, the three different commercial adjuvants tested in our study were all found to modify the elicitor activity of PSP1, including A1 and A2 sharing the same active principle, NPEs (**Figure 1**). This result indicates that the other components of the formulation of surfactant A1 such as chelating and acidifying agents negatively affected the performance of PSP1. No negative effect was observed regarding defense-eliciting activity when PSP1 was combined with the herbicide or insecticide. In fact, a somewhat increased protection against target spot was seen when PSP1 was combined with the latter. Although the insecticide was applied to plants at a non-inhibitory concentration against the pathogen *C. cassiicola* as tested *in vitro*, this observation is probably due to that the commercial insecticide possesses some antifungal activity *in situ* (over foliar surface), as was discovered when the product was tested *in vitro* against *C*. *cassiicola* (data not shown). In a second greenhouse study, the possible combinatorial use of PSP1 with important commercial fungicides used in soybean production was conducted. Results from this study showed the importance of a proper formulation and thorough testing of mixtures of agrochemicals to obtain best possible disease protection effect. When combining PSP1 with adjuvant A3 and fungicide F1, protection against target spot markedly increased (from 50 to 80%), as compared to treatment with PSP1 or fungicide alone, indicating a synergistic/complementary effect between PSP1 and fungicide when combined with A3.

Once compatibility and disease protection studies had revealed the best combinatorial product regarding elicitor efficiency, easiness of handling and straightforwardness of integration into conventional crop management, multiple field trials in central Argentina between years 2013 and 2015 were conducted. The purpose of these studies was to obtain information on PSP1 performance on crop production under normal field growing conditions. There is a big difference in plant behavior when grown in a controlled or semi-controlled environment as compared to plants grown in the field where they are continuously exposed to both biotic and abiotic challenges (Heil, 2014). It is therefore logical to assume that during the life cycle of a field grown plant, induction of plant defenses is occurring repeatedly. For this reason defense activators that have performed well when used to control pathogen infections in laboratory or greenhouse conditions have shown greater variability and less effectiveness when applied in field trials (Walters and Fountaine, 2009). For example the bacterial protein harpin, a widely studied defense-elicitor, has been found to be ineffective in controlling certain pathogens under field conditions although its efficacy in disease control had previously been confirmed under greenhouse conditions (Hopkins, 2002; Keinath et al., 2007).

Therefore, to ensure that disease-protection demonstrated under controlled growing conditions by PSP1 were reproducible under normal crop production circumstances, development of naturally occurring LSDs (FLS, SBS, and LB) was evaluated in field-grown soybean treated with PSP1 during two consecutive growing seasons at different locations in the Pampas region. An initial field trial in 2014 was followed up by multiple field trials at 14 locations varying in crop management practices and including seven different elite varieties in 2015. As was evident from our study, PSP1 is capable of inducing an enhanced pathogen defense and significantly reduces fungal disease development in field-grown soybean plants. This defense induction seems to be broad-range as disease development was clearly reduced for at least three different LSDs caused by necrotrophic fungi in these field trials, namely SBS (*S. glycines*), LB (*C. kikuchii*), and FLS (*C. sojina*). Interestingly, PSP1 produced a similar or better protection against LSDs (SBS and LB) as compared to the commercial fungicide included in the study during both years of testing. This result is one of the first examples showing an elicitor with disease control effectiveness comparable to commercial fungicides, as previously reported for Si-application that was shown to improve host plant resistance and suppress diseases as effectively as some fungicides in rice (Seebold et al., 2001, 2004).

However, this tendency observed in comparable protective effects of PSP1 and the fungicide was not completely accompanied when comparing yields, as fungicide-treated plants gave a 2.5-fold higher grain yield *per* ha than PSP1 in the 2015 trials. However PSP1 did give an interesting increase in yield (3.8%) when analyzing all 14 trials indicating its usefulness as an interesting alternative and/or complement to traditional chemical pesticides. We do not have a direct explanation for this difference in yield, although we suspect that the timing of application of PSP1 is a very important factor (see below). Notwithstanding, it is clear that the protection provided by PSP1 is complementary to the fungicide treatment as best yields were obtained by the combined application of fungicide and PSP1, demonstrating a clear additional effect as compared to the two treatments separately. This effect is probably due to that PSP1 has an important defense eliciting capability rendering its greatest protective effect prior to the onset of disease (preventive treatment), while the fungicide is applied at inhibitory doses with the aim of acting directly on the pathogen, when the disease has already manifested its first symptoms (curative treatment). Therefore, we assume that application of both products, with complementary disease protection mechanisms, mediates a better and longer period of protection against an attacking pathogen.

Another important difference to take into account is the timing of application where PSP1 was applied at growth stage V6 whereas fungicide treatment was performed at the later stage, R3 during the 2014/15 trials. Interestingly, applications of both products were performed at stage R3 in the first year of testing in Tandil accompanied by equal responses in yield increase indicating that for best results, a single PSP1-treatment should probably be performed at R3. Tests to evaluate growth parameters and biotic stress resistance at different stages of soybean development under both controlled growing conditions and field trials on different genotypes are however necessary to better define the optimal growth stage of PSP1 application.

Another critical parameter for induced disease resistance is the plant genotype as crop varieties react differently to elicitor application. Such response difference was described for the AsES protein, active component in PSP1, in strawberry (Chalfoun, 2009) where four different genotypes were challenged with the AsES-enriched supernatant and where clear differences in anthracnose disease development among varieties were observed. In the soybean field trials conducted in this study seven different elite varieties were included, indicating that although differences in receptiveness toward PSP1 is probably occurring, in the large majority of trials (24/26) improved disease resistance was demonstrated, which included six of the seven genotypes. Ample genotype responsiveness is a very important aspect for defense-induction by activators and it is desirable that induced disease resistance is tested in genetically distinct elite genotypes and breeding germplasm under both controlled and field conditions to ensure broad-range activity before commercialization.

Another major criticism of elicitor-induced crop disease management has been its low level of predictability. Application of the same elicitor significantly reduces disease and increases yield in one but not the next year, at one but not another site, in one but not another cultivar, and so on and so forth. An obvious source of variability (the most important) in the efficiency of induced disease resistance is the level of current disease pressure that depends on the crop management practices and the environmental conditions (Heil, 2014). Any type of induced resistance can benefit the plant only in the presence of the respective enemy. For example, resistance priming in barley had positive effects on yield only when high disease pressure occurred (Walters et al., 2009) and treatment of oilseed rape by a combination of BTH, BABA, and *cis*-jasmone did not benefit yield in years with low incidence of light leaf spot disease (Oxley and Walters, 2012). Therefore it is logically to assume that disease protection will only be manifested in higher yields when disease pressure is relatively high.

This postulation was corroborated in the two previously reported soybean field trials studying elicitor-induced disease protection. Abdel-Monaim et al. (2012) indicated that soybean seed soaking in an elicitor mixture consisting of BTH and humic acid, produced disease severity reductions of up to 19% for damping-off and 20% for *Fusarium* wilt, leading to simultaneously increased growth parameters and seed yield. This increase of yield was manifested in four soybean cultivars with different resistance degree sown in two different locations in Egypt and was directly proportional to disease protective effect (Abdel-Monaim et al., 2012). In another study Dann et al. (1998) described that repeated foliar applications of INA reduced severity of white mold caused by *S. sclerotiorum* by up to 50% in highly susceptible cultivars, whereas the effect was not as large in moderately resistant cultivars (Dann et al., 1998). During three consecutive growing seasons multiple INA application produced a reduction of disease severity, although this effect was accompanied by a significant increase in seed yield only when disease pressure reached 30% (Dann et al., 1998).

The aforementioned importance of disease pressure is most certainly true when there is only one major disease affecting production, but more complex to assess when there are more than one disease occurring during the growing season. From our studies it seemed that LB had a more detrimental effect on soybean yield at lower infection rates compared to SBS as 75% (3/4) of the low yield trials showed a high *C*. *kikuchii* infestation in the control plots (**Figure 5** and Supplementary Table 3). This observation is in accordance with literature that indicates that low severity levels of LB can have a relatively detrimental effect on soybean yield in the Pampas region (Carmona et al., 2010). In support of this observation, an inverse correlation between increased yield (reaching an average increase of 144 kg/ha and a maximum increase of 1,373 kg/ha in field trial E14) and reduced LB disease severity was observed. Nevertheless, a very different result was registered in trial E26 where a high degree of LB infestation but no reduction in disease severity for any of the treatments showed important yield increase. This contrasting result might be explained by the occurrence of another disease or biotic stress not monitored in this study with important yield penalty. However, this postulation does not explain the lack of effect of treatments on *C*. *kikuchii*, which could be explained by an ineffective application due to climate circumstances or an earlier or later infestation at this experimental site, before treatments or when treatment effects (both fungicide and PSP1) had disappeared.

A major concern, and a direct consequence of the importance of a high disease pressure to obtain a direct benefit from induced resistance for disease management, is the possible energy cost of the crop plant when treated with a defense elicitor. In order to enhance the resistance levels, any resource in a plant which is being allocated to the *de novo* synthesis of resistance compounds will be missing for other important physiological processes, such as photosynthesis, growth, storage and ultimately, reproduction (Herms and Mattson, 1992). As a consequence, in growth conditions with low disease pressure, application of an elicitor could therefore result in lower yields by diverting metabolic activity from biomass and/or seed development. To prevent such an allocation cost it is possible to use a lower dosage of the elicitor, which only produces a priming effect in the plant, or also to use PSP1 into some kind of slow release system to get a lower dosage PSP1 effect along the time. Priming means that a minimal induction of resistance-related genes and no increase of resistance-related compounds can be observed in a plant as long as it remains free from a pathogen attack (Perazzolli et al., 2011). However, primed plants induce their genes and/or resistance-related compounds much faster than control plants once they are being infected. Thus, primed plants invest in resistance expression only when it is required, a phenomenon that has commonly been interpreted as a cost-saving strategy (Walters and Heil, 2007).

Allocation costs have been demonstrated for soybean where jasmonic acid-treated plants produced seeds with reduced germination rates (Accamando and Cronin, 2012) and elicitor application was shown to produce reductions in growth parameters (i.e., biomass and plant height) under greenhouse conditions (Faessel et al., 2008; Srivastava et al., 2011). In this aspect it is interesting to notice that application of PSP1 did not seem to negatively affect yields when compared to control plants in our field trials. Nevertheless, we have previously observed that a double foliar application of PSP1 in some cases can give a lower protection as compared to single treatments in soybean under field conditions (data not shown), suggesting that this observation could be attributed to an energy cost for the double treated plant. In contrast, no penalizing effect on disease protection was seen in double PSP1-application in the two monocots wheat (PSP1 was applied in different crop growth stages corresponding to early tillering (Z2.1–2.3) and stem elongation (Z3.1-3.3)) and sugarcane (PSP1 applied 5 and 2 days before pathogen inoculation in plants grown under controlled conditions) (unpublished results). This difference could possibly be explained by that the recognition mechanism for some elicitors is different among plant species (differences in receptors and signaling pathways) (Qutob et al., 2006; Aslam et al., 2009) and therefore the resistance-inducing product is more effective in some plant species compared to others. As a consequence of such differences in elicitor response, a double application could be prejudicial to the growth development in some crops but not in others.

In this study, we have shown that a combination of PSP1 and a fungicide was the most effective treatment in suppressing disease development and contributed to an increase in yield. A similar effect has previously been observed in soybean treated with ASM combined with fungicides (Duarte et al., 2009). It is however very important to notice that during the development and testing of PSP1 in soybean production it has become evident that a proper agronomic evaluation in order to find an efficient and reproducible way of product application is necessary. It is obvious that for every crop species a completely new application procedure will have to be developed to ensure successful results from PSP1 treatments that include timing, number of applications, concentration and product formulation. Results from the field-trials suggest that PSP1 is an excellent complement to chemical fungicides but, can also be used as a replacement of such products for effective disease control in soybean. Additionally, it is important to note that PSP1 is cheap to produce in large amounts, easy to store and apply in the field, all important aspects for making it an interesting alternative to the more expensive chemical fungicides on the market.

In either case the implementation of a completely harmless and biodegradable product will be an important step forward to generate a more sustainable agricultural production system, and that this and similar products will be economically beneficial for both extensive and intensive crop production. These products alone will not solve all the problems and concerns regarding chemical use in agriculture production but will form a major integral part of regional breeding programs and other environmental-friendly agricultural technology developments with the aim of providing necessary tools for an efficient and economically, environmentally and socially sustainable crop production in the nearby future.

## Author Contributions

NC designed and performed the greenhouse experiments and edited the manuscript. NC, SD, SR, VD, VG, MD, EM, AC, and BW conceived and designed the experiments under field conditions and JG-M carried out these field trials in the Pampas region. SD performed a rigorous statistical analysis of data. NC, SD, EM, JG-M, and BW interpreted the results. NC, SD, and BW drafted the work and wrote the manuscript. AC revised the article. All authors approved the final version of the manuscript.

## Conflict of Interest Statement

The authors declare that the research was conducted in the absence of any commercial or financial relationships that could be construed as a potential conflict of interest.

## Footnotes

1 http://www.agritotal.com/producto/biagro-accion/

2 http://www.agritotal.com/producto/biagro-siliconado/

3 http://www.bayercropscience.com.ar/content/optimizer-maíz-y-soja
