# Supplementary material for: Elicitor-Based Biostimulant PSP1 Protects Soybean Against Late Season Diseases in Field Trials
Source: Front Plant Sci. 2018 Jun 12;9:763. doi: 10.3389/fpls.2018.00763 (PMC6006009; doi:10.3389/fpls.2018.00763)
Supplement: Supplementary file 1 [file Data_Sheet_1.docx]

**Supplementary Figure 1. Geographic localization of soybean field trials.** A, Cañada de Luque (Córdoba); B, Córdoba (Córdoba); C, Colazo (Córdoba); D, Rafaela (Santa Fe); E, San Jerónimo Norte (Santa Fe); F, San Martín de las Escobas (Santa Fe); G, Fuentes (Santa Fe); H, Gualeguaychú (Entre Ríos); I, Santa Isabel (Santa Fe); J, Fontezuela (Buenos Aires); K, General Villegas (Buenos Aires); L, Pehuajó (Buenos Aires); M, La Dulce (Buenos Aires); N, Necochea (Buenos Aires); O, Tandil (Buenos Aires).

**
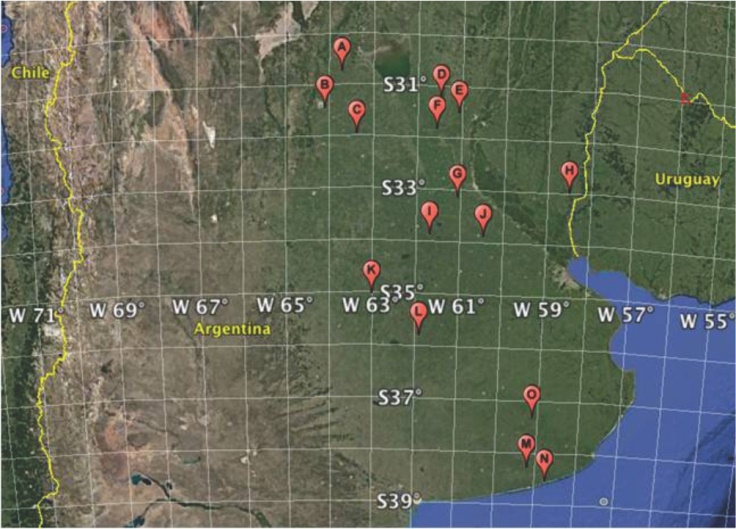
**

**Supplementary Table 1. Effect of surfactant adjuvants on the defense-eliciting activity of PSP1 against *C. cassiicola* in soybean.** Aqueous solutions of PSP1 (0.5 U ml^-1^), combined with Tween 20, A1 (Nonyphenolethoxylates (NPEs) + chelating and acidifying agents), A2 (NPEs + silicone and anti-evaporation agent) and A3 (Fatty acid methyl esters (FAME))**,** were sprayed on soybean plants grown under controlled conditions 3 days prior to inoculation with the virulent strain C4 of *C. cassiicola* and induced resistance against soybean target spot (STS) was determined. Nine biological replicates (potted plants) were assessed for each treatment and the experiment was carried out twice. Factorial ANOVA test indicated no significant differences between the two experimental repetitions (P ˃ 0.05) and results from one representative experiment are shown. STS severity was evaluated on V3 and V4 trifoliate leaves of soybean plants treated with a product or mock (pathogen control) as the percentage of leaf area covered with disease symptoms and calculated as disease severity index (DSI) at 4, 7 and 10 days post-inoculation (dpi). STS disease development in soybean plants was determined as area under the progress curve (AUDPC) from the disease severity values at different time points. Mean values of DSI at different time points and AUDPCare reported for each treatment calculated from one representative experiment with nine biological replicates. Values followed by different letters are significantly different according to Tukey’s HSD test (P < 0.05). Bold letters indicate statistically significant differences in STS protection of PSP1-treated plants as compared to mock-treated soybean plants, both infected with pathogenic strain C4 (Dunnett´s test; P < 0.05).

|  | **STS DSI(%)** | | | | | | **AUDPC** | |
| --- | --- | --- | --- | --- | --- | --- | --- | --- |
|  | **4 dpi** |  | **7 dpi** |  | **10 dpi** |  |  |  |
| Pathogen | 21.95 | b | 29.29 | b | 42.67 | b | 2.22 | b |
| PSP1 + Tween | **6.95** | **ab** | **8.25** | **a** | **14.65** | **a** | **0.59** | **a** |
| PSP1 + A1 | 7.13 | ab | 11.08 | ab | 32.92 | b | **0.94** | **ab** |
| PSP1 + A2 | **0.74** | **a** | **4.88** | **a** | **9.04** | **a** | **0.28** | **a** |
| PSP1 + A3 | 8.96 | ab | 15.78 | ab | 19.78 | ab | **1.08** | **ab** |

**Supplementary Table 2.PSP1 compatibility with commercial agrochemical products: insecticide (I), herbicide (H) and fungicide (F1) combined with different adjuvants.**The tested mixtures with adjuvant A2 are shown in (A): PSP1+A2, H+A2, PSP1+H+A2, F1+A2 and PSP1+F1+A2; and those with adjuvant A3 are shown in (B): PSP1+A3, I+A3, PSP1+I+A3, F1+A3 and PSP1+F1+A3. These mixtures were applied in soybean plants grown under controlled conditions 3 days prior to inoculation with the virulent strain C4 of *C. cassiicola* and induced resistance against soybean target spot (STS) was determined. Nine biological replicates (potted plants) were assessed for each treatment and the experiment was carried out twice. Factorial ANOVA test indicated no significant differences between the two experimental repetitions (P ˃ 0.05) and results from one representative experiment are shown. STS severity was evaluated on V3 and V4 trifoliate leaves of soybean plants treated with a product or mock (pathogen control) as the percentage of leaf area covered with disease symptoms and calculated as disease severity index (DSI) at 4, 7 and 10 days post-inoculation (dpi). STS disease development in soybean plants was determined as area under the progress curve (AUDPC) from the disease severity values at different time points. Mean values of DSI at different time points and AUDPC are reported for each treatment calculated from one representative experiment with nine biological replicates. Values followed by different letters are significantly different according to Tukey’s HSD test (P < 0.05). Bold letters indicate statistically significant differences in STS protection of product-treated plants as compared to mock-treated soybean plants, both infected with pathogenic strain C4 (Dunnett´s test; P < 0.05).

**(A)**

|  | **STS DSI(%)** | | | | | | **AUDPC** | |
| --- | --- | --- | --- | --- | --- | --- | --- | --- |
|  | **4 dpi** |  | **7 dpi** |  | **10 dpi** |  |  |  |
| Pathogen | 21.95 | c | 29.29 | c | 42.67 | b | 2.218 | b |
| PSP1 + A2 | **0.741** | **a** | **4.875** | **a** | **9.042** | **a** | **0.278** | **a** |
| H+A2 | 21.15 | bc | 30.11 | c | 47.80 | b | 2.310 | b |
| PSP1 + H + A2 | 12.04 | abc | 12.50 | b | **12.63** | **a** | **0.990** | **a** |
| F1 + A2 | **6.26** | **ab** | 10.44 | ab | **15.44** | **a** | **0.764** | **a** |
| PSP1 + F1 + A2 | **4.52** | **ab** | 9.30 | a | **18.42** | **a** | **0.713** | **a** |

**(B)**

|  | **STS DSI (%)** | | | | | | **AUDPC** | |
| --- | --- | --- | --- | --- | --- | --- | --- | --- |
|  | **4 dai** |  | **7 dai** |  | **10 dai** |  |  |  |
| Pathogen | 20.10 | b | 29.29 | b | 42.67 | b | 2.179 | b |
| PSP1 + A3 | 8.96 | ab | **15.78** | **ab** | 19.78 | ab | 1.084 | ab |
| I+A3 | 20.48 | b | 28.37 | b | 52.70 | b | 2.233 | b |
| PSP1 + I + A3 | 8.90 | ab | **12.50** | **ab** | 17.05 | ab | 1.017 | ab |
| F1 + A3 | 8.33 | ab | 17.84 | ab | 18.89 | ab | 1.126 | ab |
| PSP1 + F1 + A3 | **1.63** | **a** | **6.44** | **a** | **8.41** | **a** | **0.376** | **a** |

**Supplementary Table 3. Soybean field trials at 14 different locations in the central region of Argentina during growing season 2014-15.** Severities (%) of SBS (*S. glycines*) and LB (*C. kikuchii*) were assessed 20 and 40 days after R3 application. Trials (named E) are ranked according to decreasing severity values in the control treatment for each disease and classified according grain yield in low (L), medium M and high yield (H). Treatments were control (standard soybean crop management without foliar fungicide or biocontrol treatment), F1 (fungicide F1 alone at R3), PSP1 (PSP1 alone at V6), PSP1-F1 (PSP1 at V6 plus fungicide F1 at R3 one at the time) and PSP1+F1 (PSP1 plus fungicide F1 at R3 at the same time). Different letters indicate significant differences among treatments (according to Tukey’s HSD test; p< 0.05). Bold letters indicatetreatments significant different to the correspondent control treatment (according to Dunnet test; p< 0.05). Lower row indicates if AnoVa is significant (S) or not (NS). SBS and LB severity were not evaluated for E14 and E20, respectively. L: low yield; M: medium yield; H: high yield.

| SBS severity (%) | M | H | H |  | M | M |  | M |  | H |  | L |  | L |  | M |  | M |  | M |  | L |
| --- | --- | --- | --- | --- | --- | --- | --- | --- | --- | --- | --- | --- | --- | --- | --- | --- | --- | --- | --- | --- | --- | --- |
|  | E19 | E22 | E20 |  | E17 | E1 |  | E3 |  | E36 |  | E30 |  | E32 |  | E34 |  | E35 |  | E26 |  | E15 |
| Control | 95.5 | 81.3 | 70.5 | a | 61.3 | 56.0 | a | 55.0 | a | 47.0 | a | 29.3 | a | 23 | a | 22.3 | ab | 13.0 | a | 12.3 | a | 5.5 |
| F1 | 88.8 | 45.3 | **22.8** | b | 41.8 | 53.8 | ab | 53.3 | ab | 42.3 | a | **17.0** | cd | **18** | b | 24.0 | a | 12.0 | a | 12.0 | a | 3.3 |
| PSP1 | 89.8 | 44.0 | 36.0 | ab | 50.0 | **53.0** | b | **52.5** | b | **26.8** | b | **23.0** | b | **18** | b | **11.3** | b | 6.8 | ab | **4.0** | b | 3.3 |
| PSP1-F1 | 84.5 | 45.0 | **29.3** | b | 54.3 | **52.5** | b | **52.3** | b | **23.0** | b | **18.0** | c | **18** | b | **11.0** | b | **4.0** | b | **3.5** | b | 3.3 |
| PSP1 + F1 | 85.0 | 50.0 | **30.0** | b | 51.0 | **52.8** | b | 54.0 | ab | **27.5** | b | **14.3** | d | **17** | b | **9.8** | b | **4.3** | b | **4.0** | b | 6.0 |
| AnoVa | NS | NS | S |  | NS | S |  | S |  | S |  | S |  | S |  | S |  | S |  | S |  | NS |

| LB severity  (%) | L | L |  | M |  | M |  | L | L |  | M | M | M | M |  | M |  | H |  | H |  |
| --- | --- | --- | --- | --- | --- | --- | --- | --- | --- | --- | --- | --- | --- | --- | --- | --- | --- | --- | --- | --- | --- |
|  | E15 | E30 |  | E26 |  | E3 |  | E14 | E32 |  | E19 | E1 | E17 | E34 |  | E35 |  | E22 |  | E36 |  |
| Control | 22.5 | 21.8 | a | 19.0 | b | 17.5 | b | 16.5 | 10.5 | a | 13.3 | 13.3 | 11.5 | 11.0 | a | 8.3 | ab | 4.0 | a | 1.3 | ab |
| F1 | 25.0 | **12.3** | b | 28.0 | a | **11.3** | a | 12.8 | **4.5** | b | 8 | 10.0 | 3.5 | 9.5 | a | 10.3 | a | **0.8** | ab | 1.5 | ab |
| PSP1 | 21.0 | **13.5** | b | 19.8 | b | **12.5** | a | 13.8 | **5.5** | ab | 8.5 | 12.5 | 1.25 | **3.5** | b | **3.0** | c | **0.8** | ab | 2.3 | a |
| PSP1-F1 | 19.0 | **10.3** | b | 29.3 | a | **10.3** | a | 11.8 | 5.8 | ab | 9.75 | 7.0 | 2.25 | **3.5** | b | **4.8** | bc | **1.0** | ab | 0.5 | b |
| PSP1 + F1 | 18.8 | **11.0** | b | 29.0 | a | **12.5** | a | 7.3 | **3.8** | b | 12.5 | 8.8 | 2.25 | **1.8** | b | **3.3** | c | **0.3** | b | 0.5 | b |
| AnoVa | NS | S |  | S |  | S |  | NS | S |  | NS | NS | NS | S |  | S |  | S |  | S |  |

**Supplementary table 4. Soybean field trials at 14 different locations in the central region of Argentina during growing season 2014-15.** Yield (kg/ha) was determined for all 14 trials included in the study.Trials (named E) are ranked according to increasing grain yield values in the control treatment and classified in low (L), medium M and high yield (H). Treatments were control (standard soybean crop management without foliar fungicide or biocontrol treatment), F1 (fungicide F1 alone at R3), PSP1 (PSP1 alone at V6), PSP1-F1 (PSP1 at V6 plus fungicide F1 at R3 one at the time) and PSP1+F1 (PSP1 plus fungicide F1 at R3 at the same time). Different letters indicate significant differences among treatments (according to Tukey’s HSD test; p< 0.05). Bold letters indicatetreatments significant different to the correspondent control treatment (according to Dunnet test; p< 0.05). Lower row indicates if AnoVa is significant (S), very significant (S**) or not (NS). L: low yield; M: medium yield; H: high yield.

|  | L |  | L |  | L |  | L |  | M |  | M |  | M |  | M |  | M |  | M |  | M |  | H |  | H |  | H |  |
| --- | --- | --- | --- | --- | --- | --- | --- | --- | --- | --- | --- | --- | --- | --- | --- | --- | --- | --- | --- | --- | --- | --- | --- | --- | --- | --- | --- | --- |
| Yield (kg/ha) | E15 |  | E32 |  | E30 |  | E14 |  | E34 |  | E3 |  | E1 |  | E19 |  | E26 |  | E17 |  | E35 |  | E20 |  | E36 |  | E22 |  |
| Control | 2268 | ab | 2739 | b | 2916 | ab | 2928 | b | 3028 | b | 3065 | b | 3072 | b | 3182 | a | 3730 | b | 3801 | b | 3883 | b | 4646 | b | 4652 | ab | 4752 | b |
| F1 | 2205 | b | 3391 | ab | 3605 | a | **4033** | a | **3356** | a | 3269 | ab | 3123 | ab | 3355 | a | **4668** | a | **4068** | a | **4208** | ab | **4841** | ab | 4578 | b | **4984** | a |
| PSP1 | 2278 | ab | 2605 | b | 2675 | b | **4301** | a | 3047 | b | 3126 | b | 3394 | ab | 3210 | a | 3907 | ab | **3968** | ab | 3867 | b | 4767 | ab | 4582 | b | 4807 | ab |
| PSP1-F1 | 2362 | ab | **4084** | a | 3238 | ab | **4638** | a | 3122 | ab | **3521** | a | 3280 | ab | 3293 | a | 4287 | ab | **4003** | a | **4515** | a | **4892** | a | 4916 | a | 4944 | ab |
| PSP1 + F1 | **2715** | a | 3388 | ab | 3333 | ab | **4656** | a | 3221 | ab | 3265 | ab | **3517** | a | 3356 | a | **4673** | a | **4067** | a | **4306** | a | **4877** | a | 4787 | ab | 4929 | ab |
| AnoVa | S |  | S |  | S |  | S |  | S |  | S |  | S |  | NS |  | S |  | S** |  | S** |  | S |  | S |  | S |  |
